# Calculation model for bearing capacity of steel-CFRP composite pipeline under internal pressure

**DOI:** 10.1016/j.heliyon.2024.e27238

**Published:** 2024-02-28

**Authors:** Jianjun Shi, Wenze Wang, Wangcheng Wei, Bin Jia

**Affiliations:** Southwest University of Science and Technology, School of Civil Engineering and Architecture, Mianyang Sichuan, 621010, China

**Keywords:** CFRP, Oil and gas pipeline, Composite materials, Constitutive model

## Abstract

The Carbon Fiber Reinforced Polymer (CFRP)-wrapped steel pipeline system is emerging as an alternative for the repair and long-distance transportation of oil and gas. This system is recognized as a composite material. Under internal pressure, the pipe predominantly undergoes circumferential stretching. This study derives the tensile constitutive model of the steel-CFRP composite material using uniaxial tensile tests and the rule of mixtures. Subsequently, utilizing this newly calibrated constitutive model, a computational model to assess the internal pressure capacity of the steel-carbon fiber composite pipe was established. Finally, a comparison between the theoretical outcomes of the computational model and the actual internal pressure test results revealed a high degree of correlation. This holds substantial significance for the design and practical implementation of this novel composite pipeline system.

## 1 Introduction

Steel pipeline are the most commonly used components in the production and transportation of oil and gas industries. With the development and utilization of natural gas and submarine resources, it is expected to maintain a stable growth trend. In the future, the development of oil and gas pipeline will mainly focus on large diameter, high pressure and long-distance transportation (Frank et al., 2019) [1]. Since pipelines operate in harsh environments and transport high-pressure fluids, they may suffer various damages such as coating damage, erosion, corrosion, or mechanical damage (Kong et al., 2022) [2]. Fiber-Reinforced Polymer (FRP) performs well in both strength and corrosion resistance (Somaiah et al., 2022) [3], making it a good solution to these problems and has attracted increasing attention. In the United States, more than 60% of newly-built oil pipelines are composed of FRP. The total length of FRP pipelines exceeds 4.6 million kilometers, and it is still growing at a rate of 5%–10% annually (Scott and Scott, 2019) [4]. Currently, FRP has been used to repair and renovate deteriorated structures worldwide (Nihad et al., 2019) [5]. A reliable and economical method is to wrap continuous fibers around the periphery of the lower steel pipe to improve the peripheral pressure capacity of the pipe body and achieve the purpose of high pressure, high flow, and long-distance transportation (Alabtah et al., 2021) [6].

The unique advantages of fiber composite materials have prompted the oil and gas industry to consider their use for the development of new pipeline systems. With the expectation of achieving the most desirable physical and mechanical characteristics of both steel and FRP (Prabhakar et al., 2019) [7]. As a result, scholars have carried out some experiments on the composite of fiber composite materials and metal materials in recent years. Although studies in this area remain limited. Gang Wu (Wu et al., 2010) [8] developed a new type of steel fiber composite bar and carried out experimental research and industrial production. Fawzia (Fawzia, 2012) [9] developed an empirical model to estimate the maximum load of a given CFRP arrangement by studying the behavior of ultra-high-strength circular steel pipes reinforced with carbon fiber reinforced polymer.

So far, it has been proven that the pressure of long-distance oil and gas pipelines is borne by the steel pipe and fiber reinforcement layer when calculating the bearing capacity of the carbon fiber-wrapped metal composite pipeline structure (Sirimanna et al., 2015) [10]. Some researchers have obtained the overload pressure of steel pip and composite layer when the circumferential stress reaches the ultimate pressure capacity (Zimmerman et al., 2002) [11]. The Canadian standard CSA Z662-2015 “Oil and Gas Pipeline System” [12] stipulates the design pressure of the composite reinforcement steel pipeline. Some researchers have found that when the number of fiber layers increases, the proportion of fiber composite materials increases, resulting in an increase in the load borne by the fibers (Wang et al., 2007) [13]. However, the overall mechanical performance of the fibers will decrease. For this reason, there are certain specific process technologies and specifications, such as ISO 11439 (ISO 2000) [14] and ISO/DIS 11515.2 (ISO 2010) [15]. According to these two standards, most designers wrap oil and gas pipelines into pipelines with similar structures. So far, there is still a lack of research on fiber composite reinforced steel pipelines, a lack of engineering application experience, and no specific technical specifications. There are many issues to be resolved before the Steel-Carbon Fiber Reinforce Polymer (Steel-CFRP) composite Pipeline system can be deployed on-site.

Based on the mixture law (Yang et al., 2017) [16], the present paper firstly proposes a tensile constitutive model for Steel-CFRP composite sheet, and then conducts theoretical analysis, and establishes a calculation model for the bearing capacity of Steel-CFRP composite pipeline under internal pressure. Finally, internal pressure experiments are carried out to verify the newly proposed model. This study complements the shortcomings of the force analysis of composite pipelines, and provides the design reference and theoretical basis for using the Steel-CFRP composite pipeline as a large diameter, high pressure and long-distance oil and gas pipeline.

## 2 Tensile test

### 2.1 Overview

**Fig. 1:**
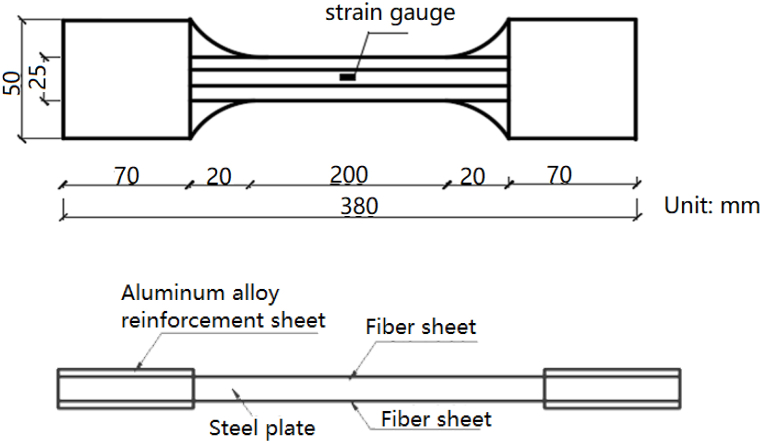
Specimen shape and size and measuring point arrangement.

**Fig. 2:**
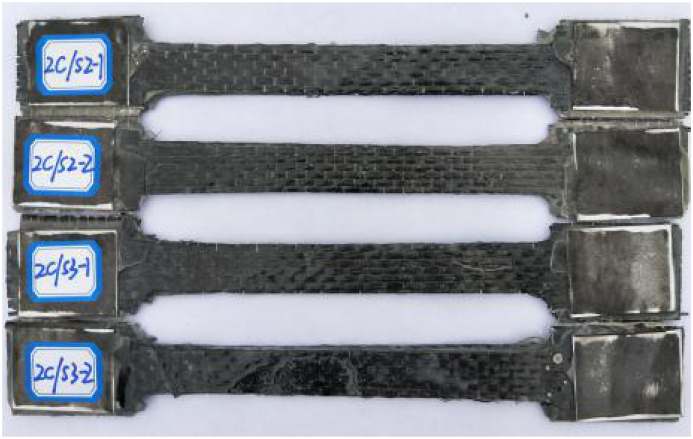
Steel-CFRP composite plate specimen for tensile test.

Tensile tests employ Steel-CFRP composite plates. The composite is fabricated by wrapping fiber fabric around a steel plate. Table 1 enumerates the properties of each constituent material. The size of the test components and the arrangement of measuring points are determined according to GB/T2010 ″Standard Test Conditions for Metallic Materials at Room Temperature” [17] and “GB/T1447-2005" [18]. Fig. 1 illustrates the specimen's shape, dimensions, and measurement point configuration. Fig. 2 displays a photograph of the Steel-CFRP composite plate specimen used in the tensile test.

**Table 1:** Material characteristics of each component of composite pipe.

Material	Elastic Modulus/GPa	Yield Strength/MPa	Tensile Strength/MPa	Elongation/%
Steel plate	192	245	360	35
CFRP fabric	240	–	3325	1.74
Adhesive	2.47	–	50.39	1.9

Tests are categorized into two groups based on the bonding of fiber fabric sheets: single-sided and double-sided. The double-sided group encompasses 13 scenarios, each differing in the number of fiber fabric layers: 1, 2, 3, or 4. Steel plates used have thicknesses of 1, 2, 3, 4, and 5 mm, respectively. Table 2 details the test condition settings for each case.

**Table 2:** Specimen number, size and CFRP laying condition in each test case.

	Number	Steel plate thickness/mm	CFRP layer number	Test piece thickness/mm	Laying angle/deg
one-sided laying	C–S3	3	1	4.35	0
	2C–S3	3	2	4.56	0
	3C–S3	3	3	5.40	0
	4C–S3	3	4	6.21	0
	8C–S3	3	8	8.67	0
double-sided laying	C/S	1	1	2.97	0
	2C/S	1	2	4.59	0
	3C/S	1	3	6.17	0
	4C/S	1	4	7.21	0
	2C/S2	2	2	5.00	0
	2C/S3	3	2	5.90	0
	2C/S4	4	2	6.94	0
	2C/S5	5	2	8.17	0

**Fig. 3:**
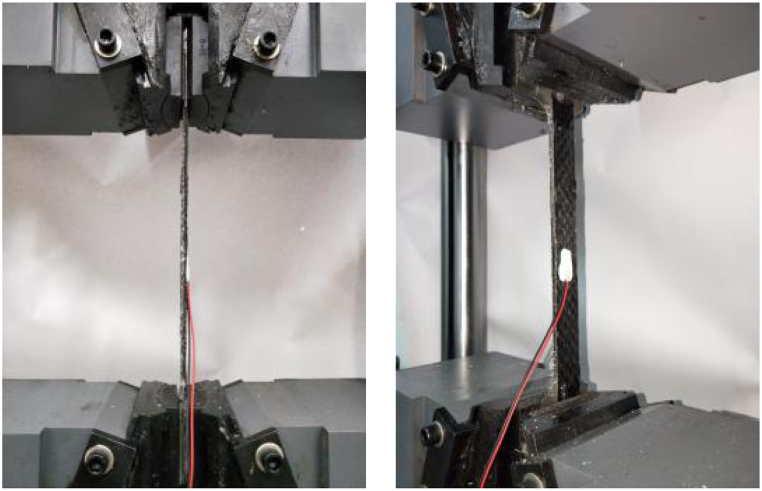
Tensile test of Steel-CFRP composite plates.

The test is carried out on a universal testing machine, loaded with the displacement control at a rate of 2 mm/min, as shown in Fig. 3.

### 2.2 Tensile test results

**Fig. 4:**
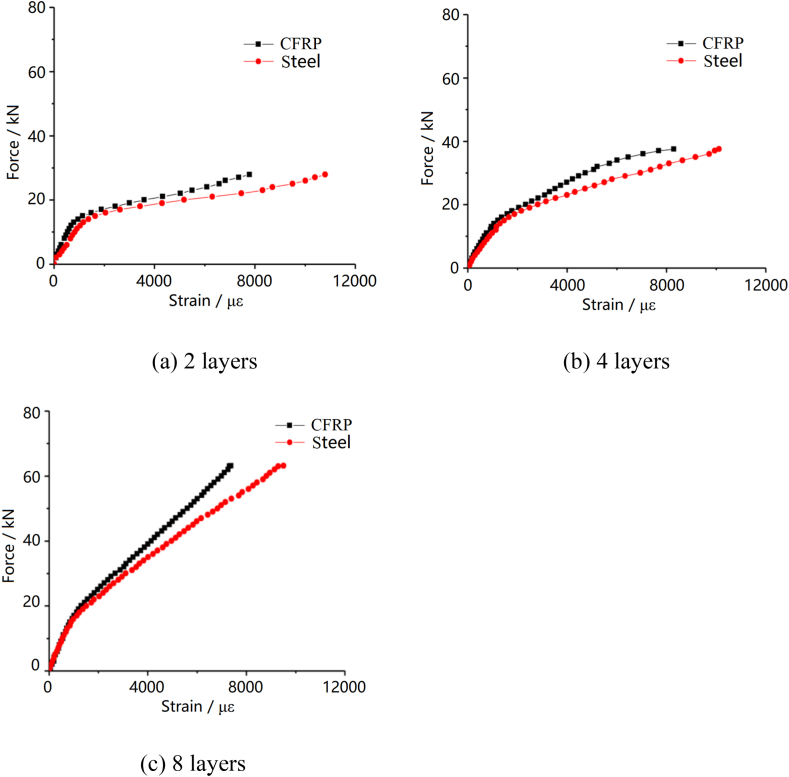
Load-strain curve for the case that CFRP fabric lay on one side of the steel plate with different number of layers.

Fracture of the fiber layer results in the failure of the test specimen. Fig. 4 shows the load-strain curve for the case that CFRP fabric lay on one side of the steel plate. The steel plate and carbon fiber fabric exhibit excellent synergistic performance. Before CFRP fabric is peeled off from the steel plate, the two materials can complement each other in mechanical properties and produce a synergistic effect to meet engineering requirements.

**Fig. 5:**
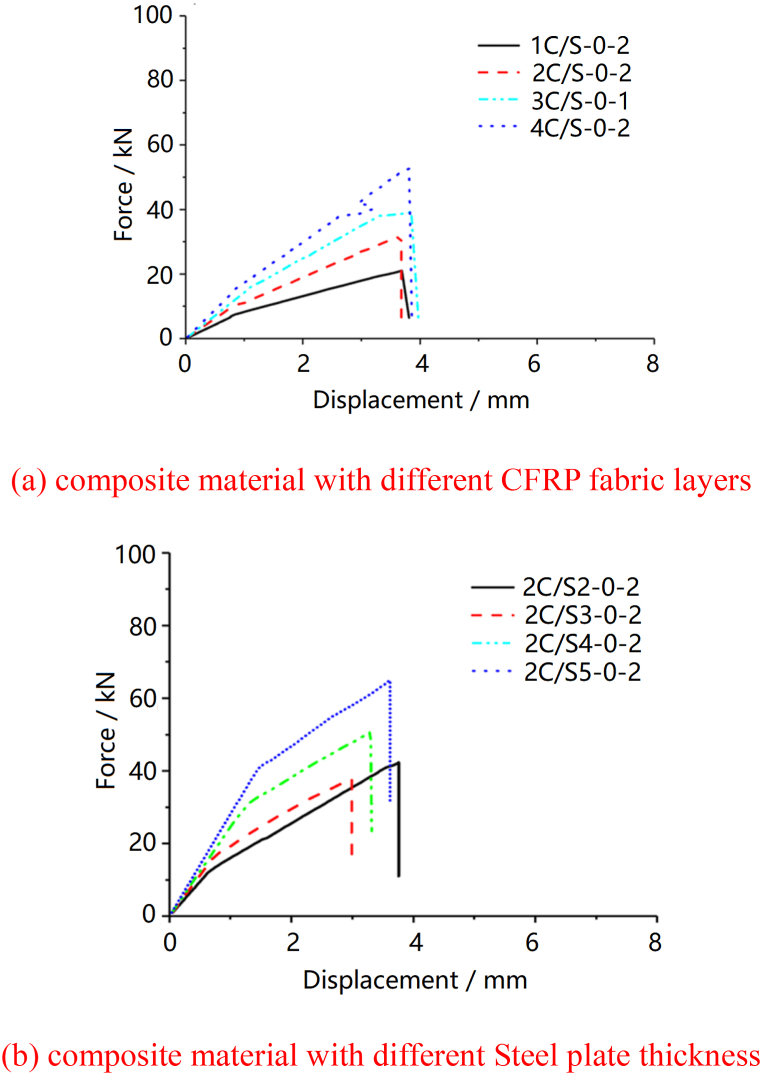
Load-displacement curve of specimens on double-sided laying CFRP fabric sheet.

Fig. 5 shows the load-displacement curves of composite sheets with different CFRP fabric layers and steel plate thickness. It indicates that the tensile stiffness of CFRP and steel composite sheet is mainly affected by the stiffness of the steel plate. The secondary stiffness of the steel plate after yielding is affected by the number of layers of carbon fiber fabric. Additionally, as the number of carbon fiber fabric layers and steel plate thickness increase, both the yield load and ultimate load of the composite plate notably escalate.

### 2.3 Tensile constitutive model

Commonly used semi-empirical models for predicting the elastic modulus of composite materials are the Voigt model (Panasenko et al., 2015) [19] and the Reuss model (Popa, 2017) [20]. The Voigt model can be expressed as:

(1) $$E_{v}=E_{m}V_{m}+E_{f}V_{f}$$

The Reuss equivalent elastic modulus can be expressed as:

(2) $$1/E_{r}=V_{m}/E_{m}+V_{f}/E_{f}$$

where $V_{m}$ is glue volume fraction; $V_{f}$ is fiber volume fraction; $E_{v}$ is Voigt equivalent elastic modulus; $E_{r}$ is Reuss equivalent elastic modulus; $E_{m}$ is the elastic modulus of the glue; $E_{f}$ is the elastic modulus of the fiber.

Prior research [21] has established the Voigt and Reuse models as the upper and lower bounds, respectively, of the elastic modulus in composite materials. Considering only one of these models will result in large distortion. In present study, the steel-CFRP composite sheet is regarded as a plate-like laminated structure composed of a CFRP sheet and a steel sheet, and the deformation of the two sheets will be coordinated and synergistic. However, CFRP sheet is composed of fiber fabric and adhesive, and the actual stress-strain state is far from the assumed state. Therefore, a scale factor α is introduced in the current work to change the elastic modulus of FRP sheets.

(3) $$E_{frp}=\alpha \left(E_{f}V_{f}+E_{m}V_{m}\right)+\left(1-\alpha \right)/\left(V_{f}/E_{f}+V_{m}/E_{m}\right)$$

where $E_{frp}$ is the elastic modulus of the FRP sheet.

According to the Mixture Law, the elastic modulus of the steel-CFRP composite sheet is:

(4) $$E_{Ι}=E_{s}V_{s}+E_{frp}V_{frp}$$

where $E_{Ι}$ is the elastic modulus of the steel-CFRP composite sheet; $V_{frp}$ is the content of the FRP sheet; $E_{s}$ is the elastic modulus of steel; $V_{s}$ is the content of steel.

Therefore, the elastic modulus of Steel-CFRP composite sheet should be:

(5) $$E_{Ι}=E_{s}V_{s}+\left[\alpha \left(E_{f}V_{f}+E_{m}V_{m}\right)+\left(1-\alpha \right)/\left(V_{f}/E_{f}+V_{m}/E_{m}\right)\right]V_{frp}$$

According to the principle of the Mixture law, the combination of two materials will produce a superimposing effect. Then, by effectively superimposing the constitutive model of steel and fiber composite material, the static tensile constitutive model of steel-CFRP composite material can be obtained.

A double polyline constitutive model is used for the steel sheet:

(6) $$\sigma =\left\{ \begin{array}{c} E_{s}\varepsilon \begin{array}{cc} & \begin{array}{cc} & 0\leq \varepsilon \leq \varepsilon_{s,y} \end{array} \end{array}\\ f_{s,y}+E_{s}^{\prime }\left(\varepsilon -\varepsilon_{s,y}\right)\begin{array}{cc} & \varepsilon_{s,y}<\varepsilon \leq \varepsilon_{s,u} \end{array} \end{array}\right.$$

where $f_{s,y}$ is the yield strength of the steel, and $\varepsilon_{s,y}$ is the yield strain of the steel; $\varepsilon_{s,u}$ is the fracture strain of the steel; $E_{s}^{\prime }$ is the slope of the second linear segment, and $E_{s}^{\prime }=\frac{f_{s,u}-f_{s,y}}{\varepsilon_{s,u}-\varepsilon_{s,y}}$, where $f_{s,u}$ is the Fracture strength of the steel.

The constitutive model of carbon fiber composite material adopts linear elastic model:

(7) $$\sigma =E_{f}\varepsilon$$

Therefore, after the steel sheet yields, the constitutive relationship of Steel-CFRP composite sheet is as follows:

(8) $$\sigma =\left\{ \begin{array}{c} E_{Ι}\varepsilon \;\begin{array}{cccc} & & & 0\leq \varepsilon \leq \varepsilon_{f,y} \end{array}\\ \sigma_{f,y}+E_{Ι}^{\prime }\left(\varepsilon -\varepsilon_{f,y}\right)\;\varepsilon_{f,y}<\varepsilon \leq \varepsilon_{f,u} \end{array}\right.$$

where $\sigma_{f,y}$ is the yield strength of FRP, and $\varepsilon_{f,y}$ is the yield strain of FRP; $\varepsilon_{f,u}$ is the fracture strain of FRP; $E_{Ι}^{\prime }$ is the slope of the second linear segment, and $E_{Ι}^{\prime }=\frac{\sigma_{f,u}-\sigma_{f,y}}{\varepsilon_{f,u}-\varepsilon_{f,y}}$, where $\sigma_{f,u}$ is the Fracture strength of FRP.

**Fig. 6:**
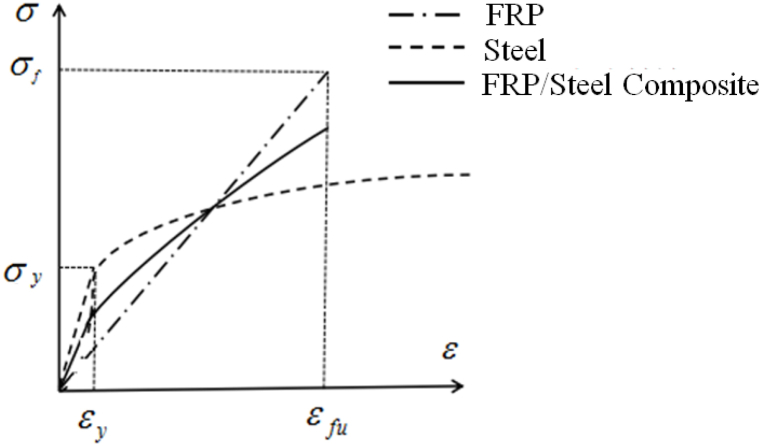
Stress-strain curve of Steel-CFRP composite based on the mixture law.

According to the mixture law, the stress-strain curve of Steel-CFRP composite is shown in Fig. 6.

## 3 Internal pressure test

### 3.1 Overview

The internal pressure test employs bidirectionally wrapped carbon fiber fabric. The Steel-CFRP composite pipeline is made of Q235 steel. The adhesive is made of epoxy resin. The length of the pipe is 1000 mm, the outer diameter is 325 mm, and the wall thickness is 5.16 mm. The mechanical properties of the pipeline are shown in Table 3.

**Table 3:** Mechanical properties of Q235 steel pressure pipeline.

Steel type	Thickness/mm	Outer diameter/mm	Elastic Modulus/GPa	Yield Strength/MPa	Ultimate strength/MPa
Q235	5.16	325	210	283	415

**Fig. 7:**
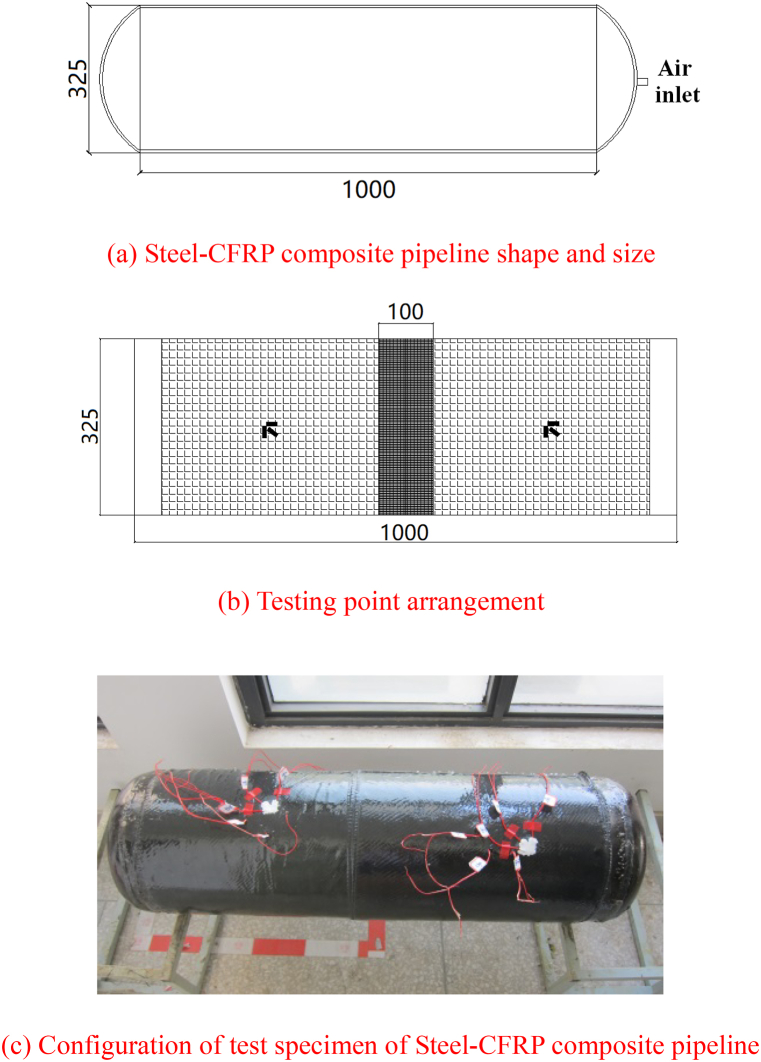
Specimen shape, size and testing point arrangement.

In order to ensure the overall mechanical performance of the pipeline, the lap length of the carbon fiber composite material is 200 mm. The experiment varies the number of carbon fiber fabric layers to assess their impact on the composite pipeline's strength. The number of layers is set to 2, 4, 6 and 8 respectively. Fig. 7 illustrates the test point configuration. Choose one section on different layers and choose two points in each section to test.

Triaxial strain rosettegage is used at each point to measure the strain along circumferential, 45° and axial direction. The strain rosettegages are attached to the steel pipe and the fiber fabric. Table 4 shows the working condition of the fiber-wrapped steel pipeline.

**Table 4:** CFRP wrapping conditions for internal pressure test.

Pipeline number	Circumferential lap size/mm	Axial overlap size/mm	Number of layers
1	200	100	2
2	200	100	4
3	200	100	6
4	200	100	8

At the beginning of the test, a pressure of 2 MPa is preloaded to check whether the instrument and test piece are working properly, and to check the air tightness of the equipment. The test adopts a staged loading system, and each stage is loaded with an additional 0.5 MPa and kept for 3 min. Gradually increase the load until the strain gauge is overloaded or the fabric breaks.

### 3.2 Internal pressure test results

**Fig. 8:**
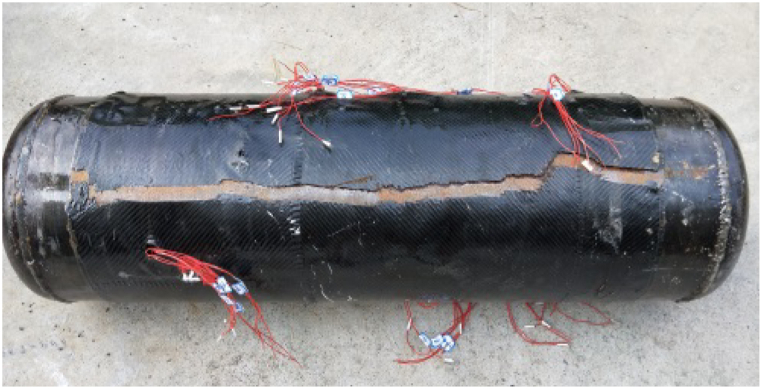
The appearance of the specimen failure.

Under normal circumstances, pressure pipelines with large diameters and long distances are mainly subjected to hoop stress. As axial stress is considerably lower, circumferential cracks may form when hoop stress attains ultimate strength. CFRP's ultimate elongation is significantly less than that of steel. Consequently, under air pressure, circumferential fracture of the carbon fiber fabric emerges as the primary failure mode, as depicted in Fig. 8.

Table 5 shows the internal pressure test results of Steel-CFRP composite pipes. It is evident that the circumferential strain in both the steel pipe and the fiber fabric significantly exceeds the axial strain, indicating that circumferential fiber plays a pivotal role in the composite pipe's bearing capacity. The yield and ultimate bearing capacities of Steel-CFRP composite pipes rise with an increasing number of fiber fabric layers.

**Table 5:** Internal pressure test results of the Steel-CFRP composite pipeline.

CFRP layer number	Yield pressure/MPa	Pressure when fiber breaks/MPa	Steel strain when fiber breaks or stop loading/με		CFRP strain when fiber breaks or stop loading/με	
			Circumferential	axial	Circumferential	axial
0	9.0	–	–	–	–	–
1	9.1	10.0	7278	3010	6540	2690
2	9.3	10.5	7044	2901	6528	2472
4	9.5	11.5	7154	2541	6750	2093
6	10.0	–	3456	1224	3316	967
8	10.5	–	2866	1065	2453	756

**Fig. 9:**
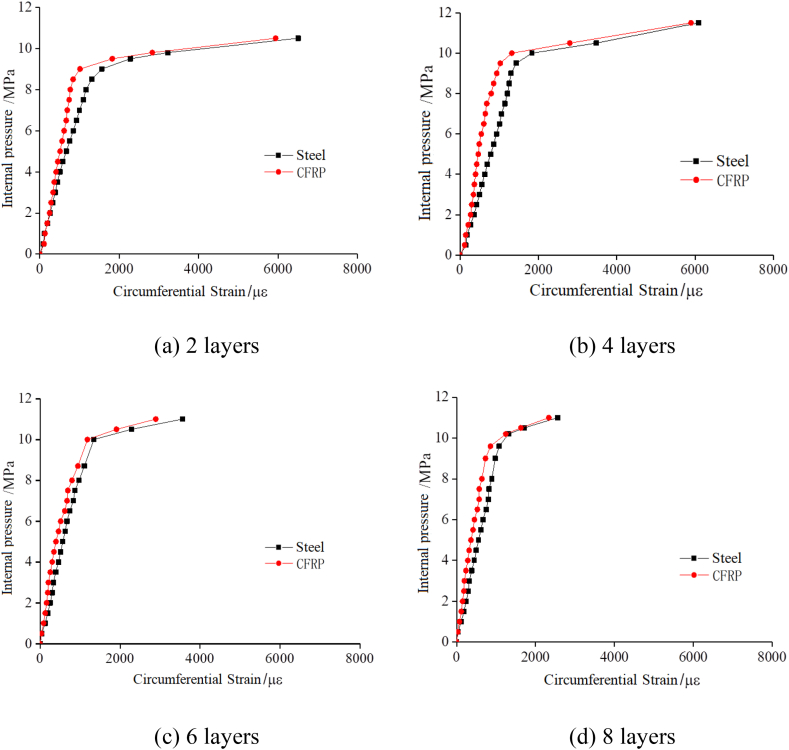
Internal pressure versus circumferential strain of Steel-CFRP composite pipeline with different number of CFRP layers.

**Fig. 10:**
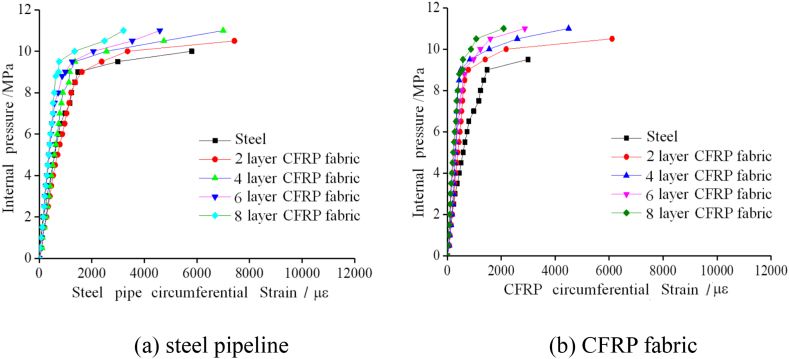
Internal pressure versus circumferential strain of steel pipe and CFRP fabric respectively with different wrapped layer number.

Fig. 9 shows the internal pressure and circumferential strain curves of composite pipes with different number of CFRP layers under internal pressure loads. Fig. 10 puts together the results of different layers in Fig. 9, in order to facilitate the comparative analysis of the circumferential strain of steel pipe and CFRP fabric as a function of the number of CFRP layers under internal pressure. It indicates that the strain of the CFRP fabric and the steel pipe increases simultaneously under the internal pressure. The load is transferred to the fiber composite material through the adhesive layer, so that the CFRP fabric can effectively share the internal pressure of the steel pipe.

As shown in Fig. 10, as the number of CFRP layers increases, the slope of the elastic phase curve increases slightly, but the slope of the elastic curve increases significantly after yielding, indicating that CFRP fabric is effective in restraining steel pipe deformation. As the number of CFRP fabric layers increases, the yield load also increases.

### 3.3 Bearing capacity model under internal pressure

**Fig. 11:**
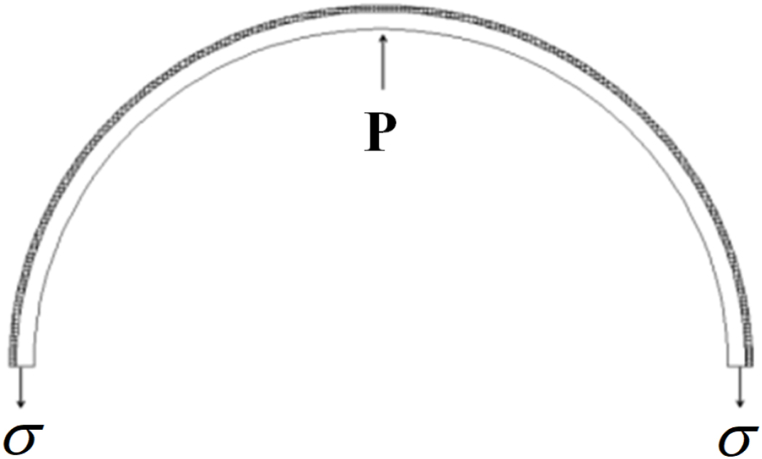
Sectional profile of the Steel-CFRP composite pipeline for theoretical analysis.

The cross-sectional profile of the Steel-CFRP composite pipeline (Fig. 11) is taken for theoretical analysis, and the constitutive relationship model is built.

The stress-strain relationship of Steel-CFRP composite under uniaxial tensile stress can be expressed as follows:

(9) $$\sigma =\left\{ \begin{array}{c} \left(E_{s}V_{s}+E_{f}V_{f}\right)\varepsilon \begin{array}{cc} & 0\leq \varepsilon \leq \varepsilon_{y} \end{array}\\ \sigma_{y}+\left(E_{s}^{\prime }V_{s}+E_{f}V_{f}\right)\left(\varepsilon -\varepsilon_{y}\right)\begin{array}{cc} & \varepsilon_{y} \end{array}<\varepsilon \leq \varepsilon_{u} \end{array}\right.$$

According to Fig. 11, the bearing capacity of the Steel-CFRP composite pipeline can be obtained as follows:

(10) $$PD_{w}=2\sigma t_{w}$$

where P is the external pressure, N; $D_{w}$ is the outside diameter of pipeline after winding fiber fabric, mm; $t_{w}$ is the wall thickness of composite pipeline, $t_{w}=t_{s}+t_{f}$; mm.

The macroscopic tensile constitutive relationship enables the calculation of the theoretical bearing capacity of the reinforced steel-CFRP composite pipeline. Because of the continuous increasing layers of winding fiber fabric, the fiber direction, the colloid distribution and the lag effect of FRP will lead to the deviation between the actual carrying capacity and the ideal carrying capacity of the pipeline. Consequently, modifications to the second stage of the bearing capacity formula are necessary as the fiber fabric layers accumulate beyond a certain threshold [22,23]. After steel pipeline yielding, most of the load will be transferred to the CFRP fabric. With the increase of load, part of the internal fibers of CFRP will break, resulting in microcracks in the adhesive layer, and the uneven force of the adhesive layer will lead to the failure to fully exert the theoretical strength of CFRP. Therefore, after entering the yielding stage, the strength reduction of CFRP should be considered. It is reported that the fiber strength of CFRP when in fracture state is only 0.4 times of the ultimate strength under the action of real internal pressure. In the pipe internal pressure test, the ultimate strain value of CFRP fabric is also about 40% of the ultimate strain of the fiber, and the steel plate remain a small ability to continue to bear the load after entering the yielding stage. In this case, the strength reduction coefficient of CFRP should be considered as fallowing:

(11) $$\sigma^{\prime }=\left\{ \begin{array}{c} \left(E_{s}V_{s}+E_{f}V_{f}\right)\varepsilon \begin{array}{ccccc} & & & & \begin{array}{cc} & 0\leq \varepsilon \leq \varepsilon_{y} \end{array} \end{array}\\ \sigma_{y}+\left(E_{s}^{\prime }V_{s}+\lambda_{f}E_{f}V_{f}\right)\left(\varepsilon -\varepsilon_{y}\right)\begin{array}{cc} & \varepsilon_{y} \end{array}<\varepsilon \leq \varepsilon_{u} \end{array}\right.$$

where, $\lambda_{f}$ is the strength reduction coefficient of the CFRP. For safer design, it takes the value as 0.3 in present study.

The revised model for calculating the bearing capacity of the Steel-CFRP composite pipeline is:

(12) $$PD_{w}=2\sigma^{\prime }t_{w}$$

**Fig. 12:**
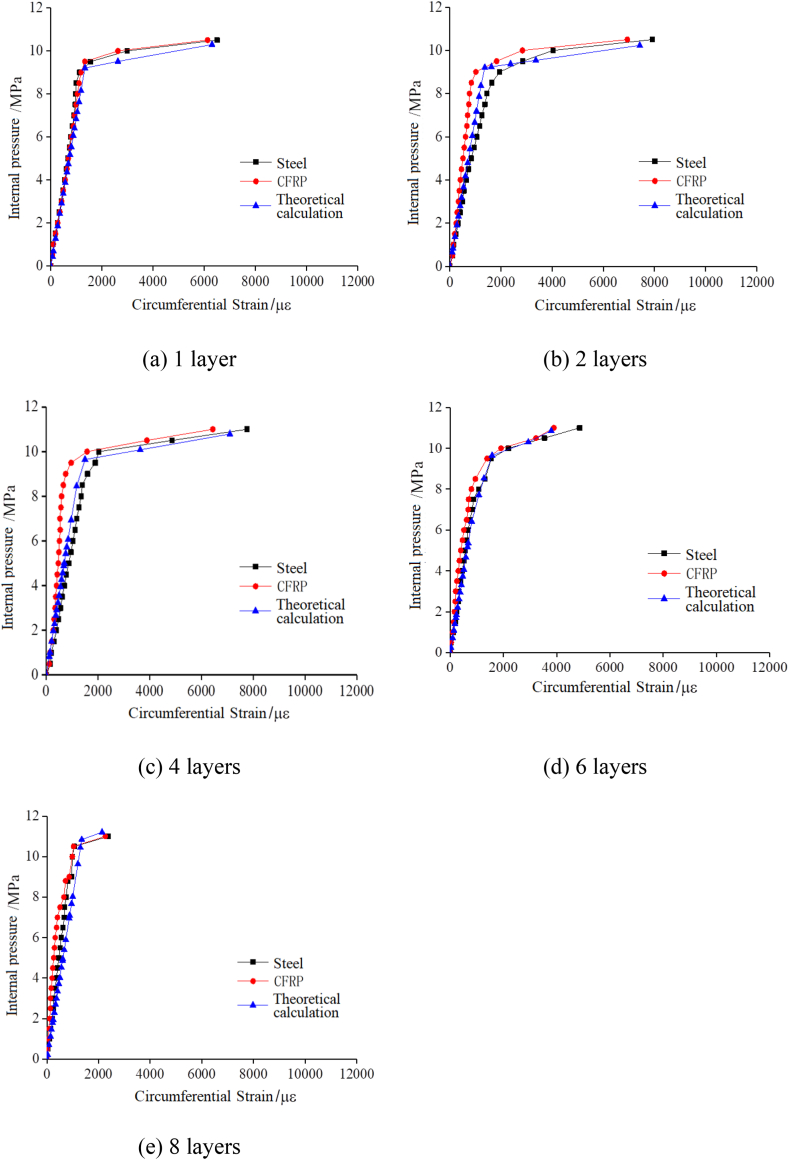
Comparison of experimental results and theoretical results.

Table 6 presents a comparison between the experimental and theoretical values of the Steel-CFRP composite pipeline's internal pressure capacity. Fig. 12 is a graph comparing the theoretical calculations with the experimental results. The results show that the yield load and ultimate load calculated according to the theoretical model are in good agreement with the test results. This validates the theoretical model's reliability in estimating the Steel-CFRP composite pipeline's bearing capacity.

**Table 6:** Comparison of theoretical calculations and experimental results.

CFRP layers	Yield pressure/MPa		Numerical difference	Internal pressure when fiber breaks or stop loading/MPa	
	experimental	theoretical		experimental	theoretical
1	9.1	9.42	4.4 %	10.0	9.93
2	9.3	9.56	3.3 %	10.5	10.36
4	9.5	9.95	4.2 %	11.5	11.32
6	10.0	10.36	3.0 %	12.0 (stop loading)	–
8	10.5	10.76	6.7 %	12.0 (stop loading)	–

## 4 Conclusions

The composite pipeline is constructed by adhesively bonding the steel pipeline with the CFRP fabric. Under internal pressure, the CFRP fabric deforms in conjunction with the steel pipeline. Throughout the loading process, these materials exhibit excellent collaborative properties, significantly enhancing the pipeline's yield bearing capacity under internal pressure. In accordance with the rule of mixtures, this paper establishes a uniaxial tensile constitutive model and a computational model to evaluate the Steel-CFRP composite pipeline's bearing capacity under internal pressure. The newly proposed models demonstrate a high degree of congruence with the experimental results.

## CRediT authorship contribution statement

**Jianjun Shi:** Writing – original draft, Writing – review & editing. **Wenze Wang:** Data curation, Formal analysis, Writing – review & editing. **Wangcheng Wei:** Validation, Visualization. **Bin Jia:** Funding acquisition, Resources.

## Declaration of competing interest

The authors declare the following financial interests/personal relationships which may be considered as potential competing interests:Jianjun SHI reports financial support was provided by 10.13039/501100018542Natural Science Foundation of Sichuan Province (Grant No. 2022NSFSC0317).
